# Antibiotic Utilization and Prophylaxis in Paediatric Cardiac Surgery: A Retrospective Observational Study at a Rural Tertiary Care Hospital in India

**DOI:** 10.7759/cureus.45107

**Published:** 2023-09-12

**Authors:** Sohilkhan R Pathan, Vishal V Bhende, Tanishq S Sharma, Amit Kumar, Vishal A Patel, Kruti B Sharma, Shivangi B Pandya

**Affiliations:** 1 Clinical Research Services (CRS), Bhanubhai and Madhuben Patel Cardiac Centre, Bhaikaka University, Karamsad, IND; 2 Pediatric Cardiac Surgery, Bhanubhai and Madhuben Patel Cardiac Centre, Bhaikaka University, Karamsad, IND; 3 Pediatric Cardiac Intensive Care, Bhanubhai and Madhuben Patel Cardiac Centre, Bhaikaka University, Karamsad, IND

**Keywords:** surgical site infections, antibiotic stewardship, healthcare, tertiary care hospital, rural healthcare, prophylactic antibiotics, antibiotic utilization, paediatric cardiac surgery

## Abstract

Introduction

Antimicrobial prophylaxis, involving short antibiotic courses preceding surgical procedures, is recommended to minimize postoperative infections. Paediatric cardiac surgeries are classified as clean procedures, though infection challenges persist due to illness severity and extended ICU stays. Antimicrobial prophylaxis varies, ranging from single doses to extended administration until catheters are removed. Typically lasting 24 to 48 hours, it has proven infection-reduction benefits. Despite these practices, uncertainties surround the optimal nature, timing, and duration of administration. This concern is amplified by escalating antimicrobial resistance driven by antibiotic overuse. Vulnerable paediatric populations bear heightened consequences of irrational antimicrobial use, contributing to global resistance trends. Yet, a defined optimal prophylaxis schedule for paediatric cardiac surgery is lacking. Importing adult guidelines may be inadequate due to paediatric research complexities and population diversity. Developing effective prophylaxis protocols is crucial for children undergoing cardiac surgery, given global antibiotic overuse and evolving drug resistance. Establishing an optimal prophylactic strategy remains a challenge, necessitating further research for evidence-based protocols to mitigate infections in this vulnerable patient cohort.

Methods

This study investigates antibiotic use in paediatric cardiac surgery. A retrospective analysis of 100 patients from a rural Indian hospital (2017-2018) assesses antibiotic patterns, including type, dose, duration, and adherence to prophylaxis protocols.

Results

In the studied cohort of paediatric cardiac surgery patients, complete compliance (100%) with antibiotic prophylaxis was observed. However, deviations were identified: 30% received antibiotics prematurely, and 30% did not align with institutional protocol criteria. Concerning antibiotic selection, 87% followed hospital policy with the recommended cefoperazone and sulbactam combination plus amikacin, while 9% received piperacillin/tazobactam + amikacin due to sepsis. Irregular use (22%) based on clinical records occurred. Furthermore, 4% received piperacillin/tazobactam + teicoplanin, with one instance of inappropriate higher antibiotic use. Regarding prophylaxis duration, only 27% adhered to the appropriate timeline, with 40% exceeding 48 hours, indicating extended use. Upon discharge, a notable proportion (45 patients) received antibiotic prescriptions. Among them, 73% were prescribed rationally, while 27% exhibited irrational antibiotic use.

Conclusion

The findings of this study shed a significant light on the issue of antibiotic misuse within the context of paediatric cardiac surgery. It underscores the pressing need for more stringent measures to regulate and address this concerning trend. The study underscores the pivotal importance of adhering rigorously to established protocols and guidelines for antibiotic prophylaxis. This adherence not only holds the potential to elevate the overall quality of patient care but also plays a critical role in combating the escalating challenge of antibiotic resistance. Through a concerted effort to optimize antibiotic usage, we can simultaneously enhance patient outcomes and contribute to the ongoing fight against the emergence of antibiotic-resistant strains, thus preserving the efficacy of these vital medications for future generations.

## Introduction

Antimicrobial prophylaxis, a very brief course of antibiotics initiated just before the start of surgical procedures, is recommended to reduce postoperative infection [1].

As a clean procedure, paediatric cardiac surgeries should have a lower risk for infection; however, given the severity of illness and prolonged ICU stays, infections remain an ongoing challenge. Antimicrobial prophylaxis in paediatric cardiac surgery takes numerous forms. Regimens vary greatly, from single-dose prophylaxis to continuing antibiotics until all chest tubes and central venous catheters have been removed. Antimicrobial prophylaxis varies usually in cardiac surgery from 24 hours to 48 hours antimicrobial prophylaxis. Antibiotic use in the perioperative period is a well-established adjunct to reducing the incidence of infection [2].

However, the nature, timing, and duration of administration remain undetermined. Further, in the context of increasing attention to antimicrobial resistance predicated upon the overuse of antibiotics, addressing this issue is timely. Paediatric populations are vulnerable to the majority of illnesses and the adverse effect of irrational use of antimicrobials is more serious among children than adults. If antibiotics are used inappropriately, it leads to the emergence and worrying of national and global trends of antimicrobial resistance. However, in paediatric cardiac surgery, an optimal schedule has not been defined. Paediatric recommendations follow the guidelines for adults, which might be improper because of the inherent challenges in paediatric research and the heterogeneity of the population. Implementation of an effective prophylaxis protocol is needed for children undergoing cardiac surgery, especially in view of worldwide antibiotic overuse and the development of drug resistance [3].

Children's metabolic immaturity poses significant challenges in the medical field, making it arduous for healthcare professionals to accurately determine the suitable dosage of antimicrobial drugs and reliably predict their pharmacokinetics and pharmacodynamics [4]. Cardiac surgery procedures pose a specific risk of infectious complications due to factors linked to extracorporeal circulation and hypothermia [5]. Paediatric recommendations for surgical site infection (SSI) management are based on experts’ opinions and data extrapolated from adult guidelines [6]. Adult guidelines are based on risk factors such as obesity, diabetes, and smoking. These items, however, are not the same risk factors identified for SSIs in children [7].

Cardiac surgery and cardiopulmonary bypass result in an immunoparalyzed state in children making them susceptible to sepsis and other hospital-acquired infections [8]. Potential pre-operative, intra-operative, and post-operative risk factors for children include age lower than one month, duration of surgery, presence of genetic abnormalities, prolonged extracorporeal circulation (ECMO) time, delayed sternal closure, pre-operative hospitalization and prolonged post-operative hospitalization, post-operative haemorrhage, and persistent low cardiac output [9].

Despite the importance of antibiotic prophylaxis during open chest management, no specific recommendations exist [10]. Finding an optimal prophylactic strategy remains a challenge, and further research is necessary to establish evidence-based protocols that can reduce the incidence of infections in this vulnerable patient population.

## Materials and methods

Study design

This research presents a comprehensive retrospective observational study conducted at the Bhanubhai and Madhuben Patel Cardiac Center, Shree Krishna Hospital, Karamsad, a prominent teaching hospital located in rural India. The study is aimed at evaluating the efficacy and rationality of antibiotic prophylaxis in paediatric cardiac surgery, which holds significant implications for patient care and outcomes in this vulnerable population. As a standard practice, ethical approval was obtained from the Institutional Ethics Committee (IEC) before the study's initiation, emphasizing the commitment to upholding ethical principles and ensuring patient privacy and safety.

The investigation encompasses a well-defined cohort of 100 paediatric patients (aged <14 years) who underwent cardiac surgery within a specific period, spanning from January 2017 to February 2018. This selection ensures a diverse representation of paediatric cardiac surgical cases, ranging from various congenital heart defects to acquired heart diseases. By including a wide range of patients, the study aims to capture the complexity and diversity of cardiac surgery in children, contributing to more robust and applicable findings.

Patient data were meticulously collected from the hospital's medical record department, employing a standardized case report form. This form was thoughtfully designed to capture essential parameters, such as patient demographics, intricate surgical details, antibiotic usage patterns, and infection-related parameters. By utilizing a comprehensive data collection approach, the study ensures that all critical factors influencing the antibiotic prophylaxis protocol's efficacy are thoroughly examined and considered.

The study's results are anticipated to provide valuable insights into the effectiveness of antibiotic prophylaxis in paediatric cardiac surgery, specifically focusing on its impact on reducing the incidence of SSIs and associated postoperative complications. By identifying potential areas for improvement, this research seeks to inform evidence-based practices and guide further efforts to optimize antibiotic prophylaxis strategies in paediatric cardiac surgery, thereby contributing to improved patient outcomes and enhancing the quality of care in this unique patient population.

Study population

The study population was defined based on specific inclusion criteria to ensure a well-defined and representative sample for analysis. Inclusion criteria encompassed paediatric patients aged up to 14 years who had undergone cardiac surgery at the esteemed cardiac department of Shree Krishna Hospital during a predetermined period, specifically spanning from January 2017 to February 2018. By focusing on paediatric patients within this age range, the study aimed to assess the impact of antibiotic prophylaxis in a vulnerable and distinct subgroup of the population, known for its unique physiological and clinical considerations.

The choice of the cardiac department at Shree Krishna Hospital as the study setting was deliberate, considering the hospital's reputation as a prominent teaching and tertiary care facility for paediatric cardiac services in rural India. This selection ensured access to a diverse and comprehensive patient pool, encompassing a wide range of congenital heart defects and acquired heart diseases that required cardiac surgery.

In contrast, to maintain the study's specificity and to ensure homogeneity in the data analysis, exclusion criteria were strictly applied. Patients above 14 years of age were excluded from the study to establish a clear focus on the paediatric population. This strategic exclusion aimed to eliminate potential confounding factors associated with age-related variations in surgical outcomes and antibiotic responses.

Furthermore, non-cardiac surgical cases were excluded from the study to ensure that the analysis remained relevant and targeted to the research question's scope. By limiting the study to paediatric cardiac surgery cases alone, the investigation could concentrate on the efficacy and rationality of antibiotic prophylaxis solely within this specific surgical context.

Collectively, the meticulous selection of the study population through well-defined inclusion and exclusion criteria ensured the integrity of the research findings, allowing for a comprehensive examination of the antibiotic prophylaxis protocol's impact on paediatric cardiac surgery outcomes. The resulting data would provide valuable insights for healthcare providers, researchers, and policymakers to optimize antibiotic prophylaxis strategies, ultimately leading to improved patient care and reduced healthcare burdens associated with surgical site infections in this vulnerable patient population.

Hospital's policy for antibiotic prophylaxis in paediatric cardiac surgery

The hospital's policy for antibiotic prophylaxis in paediatric cardiac surgery is as follows: Cefoperazone and sulbactam + amikacin are administered intravenously within 60 minutes before the start of surgery. Additionally, antibiotic treatment is continued for 48 hours after the surgery is completed.

This prophylaxis protocol has been carefully designed to minimize the risk of SSIs and promote optimal postoperative recovery for paediatric cardiac surgery patients. The timely preoperative administration of cefoperazone and sulbactam + amikacin aims to achieve therapeutic drug concentrations at the surgical site, effectively providing a protective barrier against potential microbial contamination during the procedure. By ensuring the presence of these antibiotics before the start of surgery, the hospital aims to prevent the occurrence of SSIs, which can lead to increased morbidity, prolonged hospital stays, and additional healthcare costs.

Equally crucial is the continuation of antibiotic treatment for 48 hours post-surgery. This extended prophylaxis period is essential during the early recovery phase when patients are at a heightened risk of infection. By maintaining antibiotic coverage in the immediate postoperative period, the hospital aims to support wound healing, minimize inflammation, and reduce the likelihood of SSIs. This approach aligns with best practices, as extended prophylaxis has been shown to significantly decrease the incidence of SSIs in paediatric cardiac surgery.

The selection of cefoperazone and sulbactam + amikacin as the preferred antibiotics is based on their broad-spectrum coverage against common pathogens encountered in cardiac surgical settings. The combination targets a wide range of gram-positive, gram-negative, and anaerobic bacteria, providing comprehensive protection against potential sources of infection. The choice of intravenous administration ensures rapid drug delivery and optimal bioavailability, making these antibiotics well-suited for preoperative prophylaxis.

To maintain the policy's effectiveness and evidence-based approach, the hospital adopts a multidisciplinary approach. The formulation and implementation of the antibiotic prophylaxis policy involve collaboration among cardiac surgeons, paediatricians, infectious disease specialists, and pharmacists. 

The hospital's dedication to patient safety and the proactive approach to infection prevention demonstrated through this antibiotic prophylaxis policy highlights its commitment to providing exceptional care to young cardiac surgery patients. By adhering to this well-structured protocol, the hospital aims to optimize patient outcomes, enhance postoperative recovery, and reduce the burden of SSIs on young patients and their families.

In summary, the hospital's policy for antibiotic prophylaxis in paediatric cardiac surgery, involving the administration of cefoperazone and sulbactam + amikacin intravenously before and after surgery, is meticulously designed to protect patients from SSIs and contribute to better overall outcomes in this vulnerable patient population.

## Results

Demographic and surgical characteristics of the study group are presented in Table 1. A total of eight patients fell into the age group of <30 days (neonates), 44 patients were aged between 30 days and one year (infants), 30 patients were in the age group of one year to five years, while 18 patients were in the age group of five years to 14 years (paediatrics). Out of the 100 patients, 58 had a weight greater than 5 kg, while 36 patients weighed between 2.5 and 5 kg, and only six patients weighed less than 2.5 kg.

**Table 1:** Demographic and surgical characteristics RACHS: Risk Adjustment for Congenital Heart Surgery

Characteristics	Mean	SD	Median
Age (year)	2.39	3.29	0.9
Weight (kg)	8.14	5.72	5.93
Height (cm)	78.02	25.34	70
RACHS	1.85	0.74	2
Surgery duration (minute)	89.43	39.69	83
Duration of intubation (hours)	40.62	51.07	24
Pre-operative length of stay (days)	1.93	3.10	1
Total no. of hospital stay (days)	9.58	5.61	8
Length of ICU stay (days)	3.91	4.46	2

In our study group, 30% of patients were administered antibiotics preoperatively, before the day of surgery. Among these patients, 30% did not have a justified reason for antibiotic use according to the institute's protocol (refer to Table 2). A total of 10 different types of antibiotics were utilized in patients before the day of surgery. The specific pattern of antibiotics used prior to surgery is presented in Table 2.

**Table 2:** Preoperative antibiotics

Name of Antibiotics	n=100	Rational Antibiotic n (%)	Irrational Antibiotic n (%)
Cefoperazone and sulbactam+amikacin	22	13 (59.09)	9 (40.9)
Piperacillin/tazobactam+amikacin	2	2 (100)	
Ciprofloxacin	1		1 (100)
Cefixime	1	1 (100)	
Amoxicillin clavulanate	1	1 (100)	
Meropenem	1	1 (100)	
Vancomycin and levofloxacin	1	1 (100)	
Azithromycin	1	1 (100)	
Total antibiotic used	54	35 (64.1)	19 (35.1)

All patients received intraoperative antibiotics. The most common antibiotic prophylaxis regimen was cefoperazone and sulbactam + amikacin, administered to 87% of the patients. The next most frequently used prophylaxis was piperacillin/tazobactam + amikacin, given to 9% of the patients, while piperacillin/tazobactam + teicoplanin was used in 4% of cases (Table 3).

**Table 3:** Parameters of antibiotic prophylaxis in paediatric cardiac surgery

Choice of Antibiotic Prophylaxis	Number (N=100)	Percentage (%)	Rational (%)	Irrational (%)
Cefoperazone and sulbactam+amikacin	87	87	87 (100)	
Piperacillin/tazobactam+amikacin	9	9	7 (77.7)	2 (22.2)
Piperacillin/tazobactam+teicoplanin	4	4	3 (75)	1 (25)

In 13% of cases, deviation from the institute's protocol was observed due to suspected or proven ongoing sepsis. However, upon evaluating the medical records, it was found that 23% of these cases did not actually require alternate antibiotics. It is likely that these patients were clinically unwell but did not have a bacterial infection.

In Table 4, it is shown that only 27 patients received prophylactic antibiotics for a duration of less than 48 hours. Among these 27 patients, their antibiotic treatment was justified as they had sepsis, which necessitated a change or prolonged use of antibiotics.

**Table 4:** Duration of antibiotic prophylaxis

Duration	N=100	%
Prophylaxis (≤48 hours)	27	27
Prolonged prophylaxis (>48 hours)	73	73

Additionally, out of the 27 patients who received prophylaxis for less than 48 hours, six of them developed an infection at the surgical wound. Furthermore, Table 5 indicates that none of the patients in the study were reported to have mediastinitis or urinary tract infection (UTI).

**Table 5:** Prolonged antibiotic used in sepsis, surgical site infection, and no infection

Duration	n=73	Percentage (%)	Rational n (%)	Irrational n (%)
Sepsis	27	37	27 (37)	
Surgical site infection	6	8.2	6 (8.2)	
Prolonged prophylaxis with no sepsis (>48 hours)	40	54.7		40 (54.7)

In our study group, a total of 73 patients received prolonged antibiotic prophylaxis, lasting more than 48 hours. Among these, 40 patients had no infection, indicating an irrational reason for the prolonged need, contrary to the institute's protocol. This group constitutes 40% of the study population (Table 6).

**Table 6:** Postoperative antibiotics

Name of Antibiotic	n=100	Rational Antibiotics (%)	Irrational Antibiotics (%)
Piperacillin/tazobactam	5	3	2
Ciprofloxacin	20	17 (85)	3 (15)
Cefixime	19	18 (94.74)	1 (5.26)
Amoxicillin clavulanate	2	2 (100)	
Meropenem	21	21 (100)	
Vancomycin	2	2 (100)	
Azithromycin	1	1 (100)	
Colistin	10	10 (100)	
Teicoplanin	8	8 (100)	
Total Postoperative Antibiotic Used	88	82 (93.18)	6 (6.81)

Regarding postoperative antibiotic utilization, meropenem was administered to 21 patients, vancomycin to two patients, colistin to 10 patients, and teicoplanin to eight patients. These antibiotics were given based on culture reports or clinical suspicion of sepsis, as determined by the treating intensivist. Among the positive culture results, eight patients showed multi-drug resistance, indicating a high proportion of multi-drug resistant variants.

In the study group, there were two reported cases of mortality. One patient succumbed to septic shock with multi-organ dysfunction, leading to the unfortunate outcome.

Out of the 100 patients in the study, 45 patients were prescribed antibiotics at the time of hospital discharge, indicating a considerable number of prescriptions. Among these 45 patients, 73% received antibiotics rationally, while 27% received antibiotics irrationally. Once again, fluoroquinolones were used in a high proportion (Table 7).

**Table 7:** Hospital discharge of patients with antibiotics

Name of Antibiotic	n=100	Rational of Antibiotic (%)	Irrational of Antibiotic (%)
Ciprofloxacin	23	15 (65.22)	8 (34.78)
Cefixime	17	14 (82.35)	3 (17.65)
Azithromycin	1	1 (100)	
Amoxicillin clavulanate	3	2 (66.67)	1 (33.33)
Meropenem teicoplanin colistin	1	1 (100)	
Total Antibiotic Prescribed at Hospital Discharge	45	33 (73.3)	12 (26.6)

Additionally, there was one patient who chose to leave the hospital against medical advice and transferred to a district hospital. This patient was advised to continue parenteral broad-spectrum antibiotics despite leaving the hospital early.

## Discussion

The results of this study demonstrate that antibiotic prophylaxis in paediatric cardiac surgery for the study group was 100% in adherence to the institution's protocol. However, when comparing our study with other published studies, we found that the misuse of antibiotics in paediatric cardiac surgery is still prevalent. Apostolopoulou Eleni et al. [11] evaluated 51 patients undergoing surgery and found that 94% of them received antibiotic prophylaxis inappropriately, while 96% received an excessive duration of antibiotics. In our study, the misuse of antibiotic prophylaxis was observed in 40% of cases.

Nairooz H. Al-Momany et al. [12] assessed adherence to antimicrobial prophylaxis guidelines and noted that although 100% of patients made appropriate decisions to use antimicrobial prophylaxis, only 1.7% received the recommended antibiotic of choice. Furthermore, a significant percentage of patients (39.4%) received antimicrobial prophylaxis for a duration not in accordance with guidelines. In our study, only 27% of patients received an appropriate duration of prophylaxis, while 40% of patients received prolonged prophylaxis inappropriately.

Harbarth et al. [13] compared patients receiving prophylaxis for different durations and concluded that the maximum clinical benefit of prophylaxis is achieved within 48 hours, with administration for longer durations being ineffective in further reducing infection. Similarly, in our study, we found that only 27% of patients received prophylaxis for the appropriate duration of 48 hours, whereas 40% received it for longer, which may not be beneficial.

Based on a systematic review, the Society of Thoracic Surgeons recommends not continuing antibiotic prophylaxis for more than 48 hours postoperatively [14]. In our study group, 73% of patients received prophylaxis for a duration longer than recommended.

Despite some discrepancies in data due to the retrospective nature of the study using hospital records, the study revealed that all patients received the appropriate dose of antibiotics based on their weight and creatinine clearance. Moreover, all patients received prophylactic antibiotics within 60 minutes before the skin incision, in line with the institution's guidelines.

It is concerning that 45% of patients were prescribed antibiotics at the time of hospital discharge, with 27% of them not having justification for the continuation of antibiotics on discharge. Additionally, the use of fluoroquinolones at discharge is not justified in paediatric patients [15].

The study findings highlight the need for better adherence to antibiotic prophylaxis guidelines in paediatric cardiac surgery. Similar observations of inadequate adherence to antibiotic prophylaxis for surgery have been reported from India, but there are no published Indian studies specifically addressing adherence to antibiotic prophylaxis protocols for paediatric cardiac surgery.

As this study was retrospective and conducted at a single centre, caution must be exercised when generalizing the findings to other health centres. Moreover, the cost implications of irrational antibiotic use were not part of the study's scope. The one-year duration of the study limits insights into the long-term utilization of antibiotics in this context. A more extended study could provide a better understanding of antibiotic usage patterns.

## Conclusions

In this study, we observed that antibiotic prophylaxis was consistently employed in all cases of paediatric cardiac surgery. However, a concerning pattern of significant misuse and overuse of antibiotics was evident. While similar observations have been documented in studies conducted abroad, there remains a notable gap in published research from India regarding this issue. Our study underscores the urgent need for more stringent implementation of antibiotics prophylaxis protocols in paediatric cardiac surgery to optimize patient care and safety.

The widespread misuse of antibiotics in this vulnerable patient population necessitates immediate action to mitigate the emergence of antibiotic resistance and safeguard the effectiveness of these essential medications. To achieve this, regular audits and constant reinforcement of standardized protocols are vital. By closely monitoring antibiotic utilization and adherence to established guidelines, healthcare providers can identify and address areas of concern, ensuring that antibiotics are used judiciously and in accordance with best practices.

Furthermore, this study highlights the significance of conducting more comprehensive research and multi-centric studies in India to gain deeper insights into antibiotic prophylaxis practices specific to paediatric cardiac surgery. Such efforts can yield valuable data to support the formulation of evidence-based guidelines tailored to the unique needs of the Indian patient population.

In conclusion, optimizing antibiotic prophylaxis in paediatric cardiac surgery demands a collaborative approach involving healthcare professionals, policy-makers, and researchers. By implementing robust protocols, conducting regular audits, and fostering a culture of responsible antibiotic use, we can effectively preserve the sensitivity of antibiotics and safeguard the health of young cardiac patients in India.
